# Reliability of Antemortem CT Scan Findings as an Adjunct to Autopsy in Visualizing Intracranial Hemorrhage

**DOI:** 10.7759/cureus.84928

**Published:** 2025-05-27

**Authors:** Jyoti Barwa, Anil Mittal, Sunil Khanna, Gaurav Pradhan, Rattan Singh, Ajay Kumar, Monika Tanwar

**Affiliations:** 1 Forensic Medicine, All India Institute of Medical Sciences, Bathinda, Bathinda, IND; 2 Forensic Medicine, Maulana Azad Medical College (MAMC), New Delhi, IND; 3 Radiodiagnosis, Maulana Azad Medical College (MAMC), New Delhi, IND; 4 Forensic Medicine and Toxicology, All India Institute of Medical Sciences, Bathinda, Bathinda, IND; 5 Infection Control, All India Institute of Medical Sciences, New Delhi, New Delhi, IND

**Keywords:** antemortem ct scan, autopsy, fatal head injuries, intracranial hemorrhages, non-invasive technique

## Abstract

Introduction

A number of studies have been conducted worldwide comparing postmortem CT with autopsy findings, highlighting the significance of each. However, the question arises: in countries where such facilities are not available, can diagnostic antemortem CT, performed during treatment, be used as an adjunct to autopsy? This study was carried out to compare antemortem CT scan and autopsy findings of intracranial haemorrhages in cases of fatal head injuries.

Materials and methods

The study comprised a total of 55 cases of fatal head injuries that were brought for medico-legal postmortem examination at a tertiary care hospital during a period of one year. The various intracranial haemorrhages were duly noted during the autopsy procedure and subsequently compared retrospectively with the respective antemortem CT scan findings.

Results and conclusion

In the current study, the specificity was found to be 100% for subarachnoid haemorrhage (SAH), intracerebral haemorrhage (ICH), and brain haemorrhage (BH). In contrast to the above-mentioned haemorrhages, specificity was 50% for cerebral contusions. The areas of the temporal lobe, occipital lobe, and cerebellum were poorly visualized on CT scan; hence, the sensitivity of detecting contusions in these sites was 35.7%, 40%, and 30.7%, respectively. Thus, we would like to emphasize that when antemortem CT and autopsy findings are considered together, a wider range of information can be gathered.

## Introduction

Injuries to the head and neck region are among the most common and invariably fatal regional injuries, primarily because the head is often the target of choice in assaults involving blunt trauma. Additionally, when a victim is pushed or knocked to the ground in vehicular accidents, the head is frequently the first part to strike the surface. Moreover, the brain and its coverings are particularly vulnerable to moderate degrees of blunt trauma that would rarely be lethal if applied to other areas of the body [1].

Trauma to the head can result in injuries to the underlying skull and brain, leading to extradural (EDH), subdural (SDH), subarachnoid (SAH), brainstem hemorrhages (BH), intraventricular hemorrhages (IVH), and intracerebral contusion (C) or laceration (L). Some brainstem and intracerebral contusions are primary, occurring at the site of impact or shortly afterward; others are secondary, caused by changes in intracranial pressure or bleeding into infarcts resulting from vascular damage. Additionally, extensive bruising of the scalp following blunt trauma can lead to marked swelling within the scalp layers, which may not be externally visible if located in the deeper layers of the scalp [1-3].

In radiology, the conventional X-ray technique has limitations, as it cannot detect small density differences in various soft tissues, and overlapping of structures in 3-dimensional space complicates interpretation. These limitations can be overcome by the use of CT, which promptly, accurately, and non-invasively reveals intracranial and parenchymal abnormalities in acute cranio-cerebral trauma [4,5]. Therefore, CT scanning of the head is indispensable in diagnosing various injuries during the first 48 hours after head trauma due to its availability, manageable application, relatively low cost, and prognostic value [5-7].

Autopsy, considered the reference standard for determining injuries sustained in trauma cases, can efficiently explain the number of injuries, their mechanism, and the cause of death [4]. However, radiographic images taken immediately before or during autopsy can be beneficial for better documentation or corroboration of findings. These radiographic images, captured following death in the form of postmortem computed tomography (PMCT), offer a less invasive alternative that may help address ethical concerns surrounding autopsies. PMCT does not require significant manipulation or positioning of the deceased, thereby maintaining the integrity of the scene (e.g., the subject remains in the body bag) [8]. Additionally, CT creates a permanent and objective record of detected injuries that may later serve as legal evidence in court, if required [6,8]. The raw data can also be revisited indefinitely for clinical teaching and research purposes [8].

It is well known that postmortem radiography, CT, and MRI can complement autopsy findings or, to some extent, serve as a substitute when an autopsy cannot be performed [9]. Due to the non-availability of PMCT in the present study, we have analyzed and compared autopsy and antemortem CT scan findings of intracranial hemorrhages in cases of fatal head injuries.

## Materials and methods

Study design

Descriptive observational cross-sectional study.

Study population

Unnatural death cases from a North Indian population brought for postmortem examination at a tertiary care hospital, who had undergone antemortem CT scan examination during the course of treatment.

Sample size

Fifty-five cases of fatal head injuries across all age groups and both sexes were included.

Study period

The study was conducted over a period of one year.

Exclusion criteria

Cases involving surgical intervention, massive destruction of the head, or advanced signs of putrefaction were excluded.

Study measures

Various intracranial hemorrhages were documented during the autopsy procedure by a forensic expert and subsequently compared with the respective antemortem CT scan findings noted by a radiologist. The forensic expert was blinded to the antemortem radiological findings.

Statistical analysis

Data were analyzed using SPSS statistical software version 20.0. Sensitivity, specificity, positive predictive value (PPV), negative predictive value (NPV), and overall accuracy of CT were determined, using autopsy findings as the gold standard. CT images were obtained using a Somatom Drive 256-slice MDCT machine by Siemens Healthineers. The technical specifications of the machine included a current of 650-750 mA and a voltage of 70-140 kV. The slice thickness was 6 mm, with the ability to reconstruct at 1 mm. The obtained Digital Imaging and Communication in Medicine (DICOM) images were reviewed and reconstructed using syngo.via software and subsequently converted into 3D images.

## Results

Out of the 55 cases studied, males outnumbered females, with a male-to-female ratio (M:F) of 5.7:1. The age of subjects ranged from 12 to 76 years, with the highest number of cases belonging to the 31-40 years age group. The mean age was 36.93 ± 14.81 years. The majority of cases had sustained injuries in road traffic accidents (n = 41; 75%), followed by falls from height (n = 6; 11%), and assault (n = 3; 5%). In 5 cases (9%), the mode of injury was unknown. Among those who succumbed to road traffic accidents, most belonged to the 41-50 years age group, while falls from height occurred primarily in the younger age group of 11-20 years. The data obtained were analyzed using SPSS statistical software version 29.0, and the following diagnostic parameters were calculated for CT scan findings, taking autopsy results as the gold standard (Table 1).

**Table 1:** Showing the sensitivity, specificity, PPV, NPV, and accuracy. EDH: Extradural Hemorrhage; SDH: Subdural Hemorrhage; SAH: Subarachnoid Hemorrhage; ICH: Intracranial Hemorrhage; L: Lobe; BH: Brainstem Hemorrhage; IVH: Intraventricular Hemorrhage; TP: True Positive; FP: False Positive; TN: True Negative; FN: False Negative; Sn: Sensitivity; Sp: Specificity; PPV: Positive Predictive Value; NPV: Negative Predictive Value; Acc: Accuracy.

S. No.	Findings	Location	TP	FP	TN	FN	Sn (%)	Sp (%)	PPV (%)	NPV (%)	Acc (%)
1	Scalp H	-	13	0	3	39	25	100	100	7.14	29.09
2	EDH	-	4	1	41	9	30.77	97.62	80	82	81.82
3	SDH	-	34	2	12	7	82.93	85.71	94.44	63.21	83.64
4	SAH	-	39	0	1	15	72.22	100	100	6.25	72.73
5	ICH	-	13	0	30	12	52	100	100	71.43	78.18
6	Contusion	-	30	3	3	19	61.22	50	90.91	13.64	60
		Frontal lobe	25	2	12	16	60.98	85.71	92.59	42.86	67.27
		Parietal lobe	8	5	32	10	44.44	86.49	61.54	76.19	72.73
		Temporal lobe	15	3	10	27	35.71	76.92	83.33	27.03	45.45
		Occipital lobe	4	0	45	6	40	100	100	88.24	89.09
		Cerebellum	4	0	42	9	30.77	100	100	82.35	83.64
7	BH	-	6	0	37	12	33.33	100	100	75.51	78.18
8	IVH	-	5	5	42	3	62.5	89.36	50	93.33	85.45

The findings for different variables were computed using the following:

Sensitivity (Sn) = TP / (TP + FN) × 100; Specificity (Sp) = TN / (TN + FP) × 100; Positive Predictive Value (PPV) = TP / (TP + FP) × 100, Negative Predictive Value (NPV) = TN / (TN + FN) × 100; Accuracy (Acc) = (TP + TN) / (TP + FP + TN + FN) × 100.

We found that the sensitivity of all variables was below 85%, with the lowest being for scalp findings (25%, n = 13). Specificity for all variables, except for contusion, was above 50%. The CT scan showed the highest specificity (100%) for scalp injuries, SAH, ICH, and BH, indicating that these findings, when detected on CT, are highly reliable. Since a p-value ≤ 0.05 is considered statistically significant, in our study, most variables were found to be statistically insignificant. Thus, we can conclude that SDH and cerebral edema demonstrated good agreement between autopsy and CT findings; ICH, IVH, and BH showed moderate agreement; EDH showed fair agreement; while scalp findings, SAH, and contusion showed poor agreement (Table 2).

**Table 2:** Kappa values of individual variables. Strength of agreement based on Kappa statistical values: <0.20: Poor; 0.21-0.40: Fair; 0.41–0.60: Moderate; 0.61-0.80: Good; 0.81-1.00: Very Good; 1.00: Perfect.

Variable	Kappa Statistic Value	P-value
Scalp	0.035	0.322
Epidural Hemorrhage (EDH)	0.36	0.002
Subdural Hemorrhage (SDH)	0.641	0
Subarachnoid Hemorrhage (SAH)	0.086	0.115
Intracerebral Hemorrhage (ICH)	0.542	0
Contusion	0.052	0.596
Edema	0.603	0
Brainstem Hemorrhage (BH)	0.402	0
Intraventricular Hemorrhage (IVH)	0.47	0

## Discussion

In our study, CT scan was found to be insensitive for detecting EDH, with sensitivity being 30.77%, and specificity, PPV, and NPV being 97.62%, 80%, and 82% respectively. This is in concordance with Añon J et al. [7] and Molina DK et al. [10], who observed a sensitivity of 26% and 33% respectively, and a high specificity of 99% each, similar to what we obtained in our study. However, Panzer S et al. [11] observed a high sensitivity and specificity, wherein a follow-up antemortem CT after a primary CT scan was performed. Discrepancies were observed for most of the assessed pathological entities between the two scans; such comparison was not done in our study. Sensitivity and specificity for the detection of SDH in our study were 82.93% and 85.71%, respectively. This is consistent with the findings by Añon J et al. [7], Panzer S et al. [11], Kuruc R et al. [12], and Hlahla MI and Selatole MJ [13]. Although the sensitivity obtained by Molina DK et al. [10] was lower compared to ours, among the different extra-axial haemorrhages, he found that CT scan showed the best sensitivity (65.8%) for SDH. CT scans were found to be reliable in detecting SAH in our study, with a sensitivity of 72.22%, specificity of 100%, PPV of 100%, and NPV of 6.25%. It was able to correctly detect the injuries found during autopsy with an accuracy of 72.73%. This, however, is inconsistent with the findings of Molina et al. [10], Hlahla MI and Selatole MJ et al. [13], Kibayashi K et al. [14], Spiegel SM et al. [15], and Chute DJ and Smialek JE [16]. In our study, EDH showed fair agreement, while SAH showed poor agreement between the autopsy and CT findings, which is consistent with the findings of Kuruc R et al. [12] and Hlahla MI and Selatole MJ et al. [13], who reported the least discrepancy in the findings of EDH. Out of the 25 cases in our study that showed ICH during autopsy, CT scan was able to correctly detect the lesion in only 13 cases, yielding a sensitivity of 52%, while the specificity was 100%. This could possibly be due to the appearance of secondary haemorrhage occurring during the hospital stay; hence, the CT performed at presentation in the hospital did not reveal the same. However, we have not come across any literature focusing on the comparison of this type of haemorrhage. As per the study conducted by Molina DK et al. [10], CT scan is not sensitive in detecting cerebral contusions (32% sensitivity) or lacerations (0% sensitivity). However, we considered these findings together and found them to be consistent with their study, having a sensitivity of 61.2%. We also found that these types of injuries are mostly located in the frontal and temporal lobes, which is in concordance with Goyal MK et al. [17]. This finding is inconsistent with Panzer S et al. [11], who found a relatively high sensitivity and specificity of >85% for cortical contusion in their study. Out of the total 55 cases studied by us, CT could detect only 4 cases of cerebellar contusion as opposed to 13 found during autopsy, which is also consistent with Goyal MK et al. [17], who hypothesised that this failure in detection could be due to a technical fault in planning the scan. We agree with the same; in addition, the visualization of cerebellar lesions poses a problem due to their deeper location. The sensitivity of BH and IVH was low (33.3% and 62.5%, respectively), while the specificity was found to be high (100% and 89.36%, respectively). Of the total number, BH were detected in only one-third of the cases by CT, i.e., only 6 by CT as opposed to 18 by autopsy, which is consistent with Goyal MK et al. [17], Tsai FY et al. [18], and Rosenblun WI et al. [19]. This finding is inconsistent with that of Panzer S et al. [11], who found a high sensitivity for IVH by CT. Hence, it may be postulated that these haemorrhages are difficult to visualize on CT scan, particularly the smaller lesions, due to artefacts in the posterior fossa image [18,19].

The study has some limitations, as it considers only a small sample size for determining the reliability of antemortem CT scans in visualizing intracranial haemorrhages. Hence, it should be extended to involve more cases for better accountability. Additionally, since the CT scans were undertaken at the time of the patient’s arrival, followed by a considerable hospital stay until the patient’s death, secondary haemorrhages may have appeared due to cerebral oedema, which were not taken into account in the analysis.

## Conclusions

In current study the specificity is 50% for contusions which implies that in about half of the cases there are chances that it may not be present at all. There is difficulty in detecting contusions present in the temporal and occipital lobes on CT with a very low sensitivity of 35.7% and 40%, respectively. Also, the cerebellar contusions being difficult to visualize have an extremely poor sensitivity of 30.7%.

Thus, we would like to emphasize that in developing countries where facility of postmortem CT scan is not feasible, even antemortem CT findings can be a useful and reliable tool. These findings when considered together, a wider range of information can be gathered. It is a valuable piece of information not only to the autopsy pathologist in better documentation of findings but also for the physician/surgeon to retrospectively analyze the case he treated when the patient was alive.
